# Supplementary material for: Effect of Growth Stage on Nutrition, Fermentation Quality, and Microbial Community of Semidry Silage from Forage Soybean
Source: Plants (Basel). 2024 Mar 6;13(5):739. doi: 10.3390/plants13050739 (PMC10934759; doi:10.3390/plants13050739)
Supplement: Supplementary file 1 [file plants-13-00739-s001.zip › plants-2864821-supplementary.pdf]

Supplementary materials

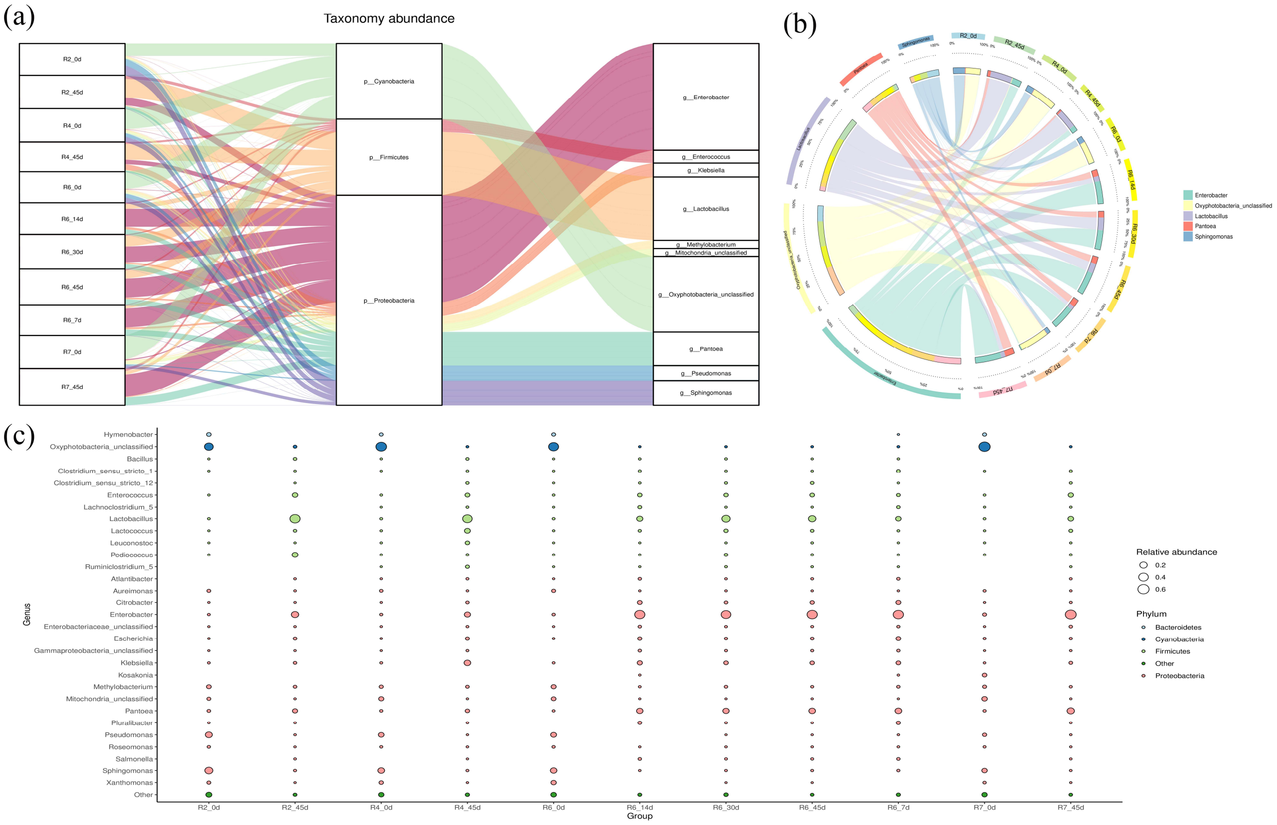

Figure S1. (a) Sankey plots of soybean haylage flora in different periods of mowing and different fermentation stages. (b) Circos circle diagram of soybean haylage flora at different cutting stages and different fermentation stages. (c) Bubble plot of soybean haylage flora at different cutting stages and different fermentation stages.

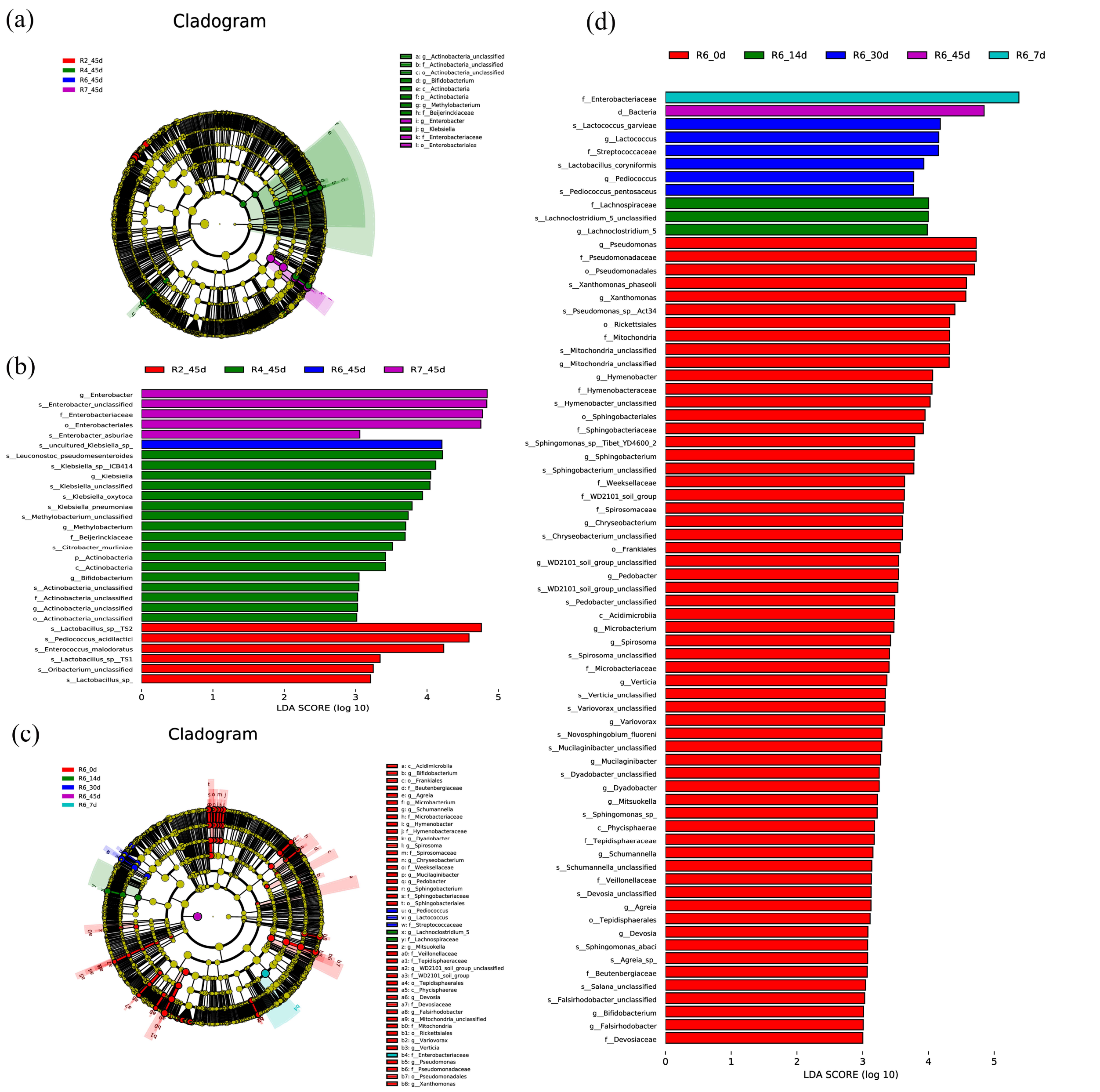

Figure S2. (a,b) Cladogram and bar chart based on LEfSe analysis of soybean haylage flora in different periods. (c,d) Cladogram and bar chart based on LEfSe analysis of soybean haylage flora at different fermentation times.

Note: The different circle layers in the figure radiate from inside to outside, representing seven taxonomic levels of the phyla, family, genera and species. Each node represents a species classification at that level. The higher the species abundance, the larger the node, the following figure is consisitent.

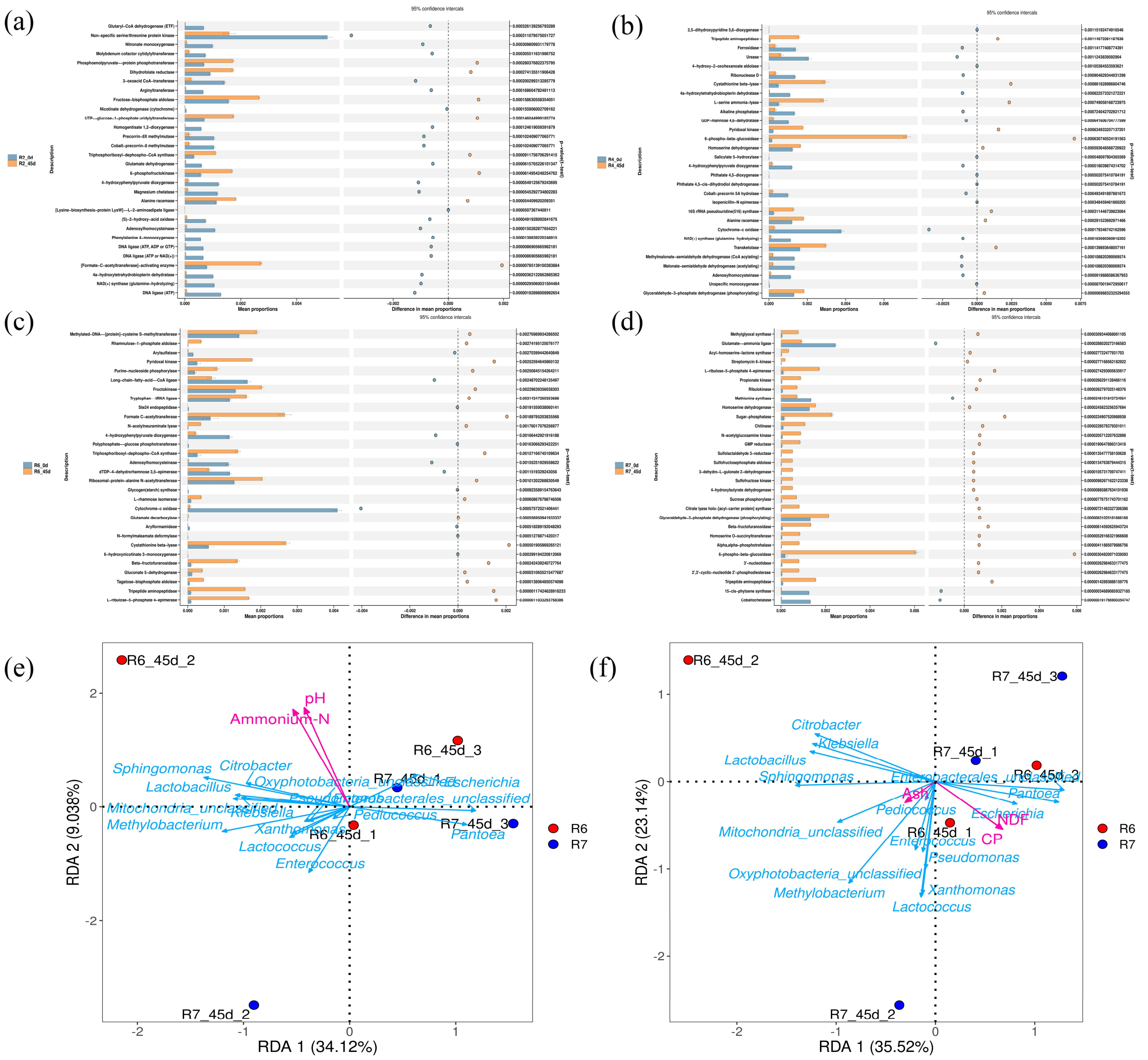

Figure S3. (a – d) Using PICRUSt2 to predict pathway function based on the EC pathway database before and after fermentation. (e,f) Redundancy analysis (RDA) based on the relationship between major microbial community and silage quality.
